# Impact of Perinatal Coexposure to Chlorpyrifos and a High-Fat Diet on Kisspeptin and GnRHR Presence and Reproductive Organs

**DOI:** 10.3390/toxics11090789

**Published:** 2023-09-19

**Authors:** Marwa Lahimer, Narimane Djekkoun, Sophian Tricotteaux-Zarqaoui, Aurélie Corona, Isabelle Lafosse, Habib Ben Ali, Mounir Ajina, Véronique Bach, Moncef Benkhalifa, Hafida Khorsi-Cauet

**Affiliations:** 1 PERITOX-(UMR-I 01), UPJV/INERIS, UPJV, CURS, Chemin du Thil, 80025 Amiens, France; marwa.lahimer@etud.u-picardie.fr (M.L.); djekkoun.narimane@gmail.com (N.D.); tricotteaux.s@outlook.fr (S.T.-Z.); aurelie.corona@u-picardie.fr (A.C.); veronique.bach@u-picardie.fr (V.B.); benkhalifa.moncef@chu-amiens.fr (M.B.); 2 ART and Reproductive Biology Laboratory, University Hospital and School of Medicine, CHU Sud, 80025 Amiens, France; 3 Exercise Physiology and Physiopathology: From Integrated to Molecular “Biology, Medicine and 9 Health” (Code: LR19ES09), Sousse 4002, Tunisia; mounirajina09@gmail.com; 4 MP3CV—UPJV—UR 7517, Jules Verne University of Picardie, 80025 Amiens, France; i.lafosse@u-picardie.fr; 5 Laboratory Histology Embryology, Faculty of Medicine Sousse, University of Sousse, Sousse 4000, Tunisia; alihabibsaoudite@yahoo.fr; 6 Service of Reproductive Medicine, University Hospital Farhat Hached, Sousse 4000, Tunisia

**Keywords:** chlorpyrifos, high-fat diet, Kisspeptin, GnRHR, ovary, testis, rat

## Abstract

Emerging evidence has indicated the involvement of extrahypothalamic Kisspeptin and GnRHR in reproductive function. In this study, we evaluate if maternal exposure to the pesticide chlorpyrifos (CPF) and/or a high-fat diet (HFD) has an impact on the expression of Kisspeptin and GnRHR in the reproductive organs of rats’ offspring. A total of 16 pregnant rats are divided into four groups: a control group (*n* = 4), CPF group (4 rats exposed daily to 1/mg/kg/day), HFD group (4 rats randomly fed a 5.25 kcal/g HFD), and coexposed group (4 rats exposed to CPF and HDF). At postnatal development postnatal day (PND) 60, male and female offspring were sacrificed. The reproductive organs (ovary and testis) were removed, and histological and immunohistological analysis and in silico quantification (TissueGnostics software 6.0.1.102, TissueFAXS, HistoQuest) were applied to investigate the impact of different treatments on Kisspeptin and GnRHR expression in reproductive organs. The main outcomes of the study showed a significant decrease in rat offspring’s body weight in the CPF group from PND30 and PND60 (*p* < 0.05 and *p* < 0.01, respectively). Histological analysis showed a significant increase in the atretic follicle and abnormal testis structure with germ cell desquamation in the CPF-exposed group. The immunodetection quantification of protein shows a significant decrease in GnRHR and Kisspeptin in the HFD and CPF exposed groups, respectively, in testis rat offspring. Perinatal exposure to CPF and HFD exposure affect the reproduction function of rat offspring.

## 1. Introduction

The World Health Organization defines infertility as the inability to achieve a successful pregnancy within 12 months of unprotected intercourse [1,2]. It affects approximately 17 to 20% of couples worldwide [2]. According to a 2015 epidemiological study, the World Health Organization estimates that nearly 186 million individuals worldwide are affected by infertility globally [3]. Numerous factors can affect fertility, including environmental and lifestyle factors such as smoking, excessive alcohol intake, and obesity [4].

Since the last decade, the literature reported that exposure to pesticides and the issue of obesity have emerged as pressing public health concerns. They are interconnected and have been associated with various adverse effects, including a decline in reproductive health and a detrimental impact on the effectiveness of assisted reproduction technology [5]. The mechanisms behind this relationship are not yet fully elucidated, but it is believed that pesticide exposure and a high-fat diet disrupt metabolic processes [6] and alter reproductive health [7]. Our previous studies have shown that pesticide exposure alters some sperm parameters including motility and vitality and increases sperm DNA fragmentation [8]. The Anses (L’Agence nationale de sécurité sanitaire de l’alimentation, de l’environnement et du travail) and EPA (United States Environmental Protection Agency) reported that certain pesticides are considered an endocrine disruptor, including the chlorpyrifos polychlorinated bisphenyls (PCB), bisphenol A (BPA), dibutyl phthalates (DBP) and alkyl phenols, Di-(2-ethylhexyl) phthalate (DEHP), Glyphosate, Phthalates, dichlorodiphenyltrichloroethane (DDT), Methoxychlor, and chlorpyrifos (CPF) [9,10].

Chlorpyrifos is an organophosphate insecticide widely used in agriculture to control pests on crops [11]. However, studies raised concerns about its potential effects on human health, including reproductive toxicity, which includes both male and female reproductive organs as well as the development of embryos and fetuses [12,13]. CPF is an insecticide that disrupts the nervous system by inhibiting the activity of an enzyme called acetylcholinesterase (AChE) [14,15], which plays a critical role in transmitting nerve signals in target insects, and it can also affect non-target organisms, including humans [16]. The developmental origins of health and disease (DOHaD) hypothesis suggests that environmental exposures and experiences during early life, particularly during fetal development and early childhood, can have a profound impact on an individual’s lifelong health and disease risk and can lead to long-term health consequences [6,17,18]. The main aspects of DOHaD include fetal programming [19], critical windows of development [20], epigenetic changes [21], long-term health consequences [22], interactions with genetics, and transgenerational effects [23]. Guidelines and regulations set by authorities like EFSA (European Food Safety Authority) and EPA (United States Environmental Protection Agency) regarding the use of pesticides like chlorpyrifos take necessary precautions to ban exposure, especially for pregnant women and young children [11,24].

Emerging evidence indicates that endocrine disruptors alter the synthesis and functions of androgens and estrogens, which are essential sex hormones responsible for the development and maintenance of the male reproductive system. It can disturb the hypothalamic and pituitary (HPG) axis function by altering the synthesis of hormones regulating reproduction, including GnRH and Kisspeptin [25].

Kisspeptin, a neuropeptide with 145-amino acid, is encoded by the KISS1 gene. It is considered a central regulator of the hypothalamic–pituitary–gonadal (HPG) axis [26]. It plays a crucial role in the regulation of reproductive function. It acts as a potent stimulator of the release of gonadotropin-releasing hormone (GnRH) in the brain [27]. GnRH serves as the key mediator, conveying these cues throughout the HPG axis [28]. Over the past twenty years, significant research has been dedicated to studying the Kisspeptin (KISS1) neurotransmitter, and a growing body of evidence supports their involvement in regulating the secretion of GnRH and gonadotropin hormones throughout the ovulatory cycle [29].

It was believed that Kisspeptin neurotransmitters are only found in the hypothalamus. However, emerging evidence revealed interesting findings indicating that Kisspeptin-producing cells are also present in several extrahypothalamic regions of the brain, as well as in peripheral tissues such as the placenta, ovary, uterus, testis [30,31], and gastrointestinal tract. It was detected for the first time in rat tissue [32,33,34,35]. It is implicated in the regulation of puberty onset, fertility, and pregnancy [36]. Extra hypothalamic Kisspeptin has been found to modulate the secretion of reproductive hormones, influence the development and function of reproductive organs, and participate in the establishment of reproductive behaviors [37,38]. Siminara et al., 2003 have reported that mutation in Kisspeptin receptor knockout (KISS1r^−/−^ or GPR54^−/−^) in mice leads to idiopathic hypogonadotropic hypogonadism (small testes in male mice and an absence of follicular maturation in female mice) [39]. Furthermore, Feng et al., 2021 have reported that the expression of the KISS-1/GPR54 system in the testes of rats is influenced by a high-fat diet, leading to a decrease in the expression level of the KISS-1 protein [30]. A case–control study published by Abdelkareem et al., 2023 included a total of 30 patients (10 patients with unexplained recurrent pregnancy loss, 10 patients with pregnancy loss due to aneuploidy, and 10 patients for the control group). The study investigated the role of KISS1 and KISS1R in early placentation. The outcome revealed a significant decrease in KISS1R expression in the chorionic tissues of (euploid and aneuploid) those with recurrent pregnancy loss compared with the control group [40].

Several studies reported that GnRHR was found not only in the hypothalamus but also in various extrahypothalamic tissues, including the ovary, oviduct [41], testis [42], mammary gland [43], placenta, and endometrium [44]. However, the specific mechanisms and the exact function of Kisspeptin and GnRHR of extrahypothalamic origin in mammals remain unknown and are still under investigation. Additional research is required to better understand the relationship between environmental factors, diet, and the implication of Kisspeptin in reproductive function.

The primary aim of this research is to identify the presence of Kisspeptin and GnRHR expression within the reproductive organs of rats’ offspring using immunohistochemistry and to evaluate the impact of exposure to chlorpyrifos and a high-fat diet on the expression of these proteins through in silico quantification. Furthermore, the study investigates the morpho-pathological effects of these exposures on the reproductive organs using histological analysis.

## 2. Materials and Methods

### 2.1. Chlorpyrifos Preparation and High-Fat Diet

Chlorpyrifos, O, O-diethyl-O-(3,5,6-trichloro-2-pyridinyl) phosphorothioate, was purchased from LGC Standards (Molsheim, France). A quantity of 250 mg of Chlorpyrifos was combined with 25 mL of rapeseed oil (MP Biomedicals, Illkirch, France) as a vehicle, resulting in a concentration of 1 mg/mL. This mixture was then administered to the rats (the CPF group and the coexposed group) via gavage at a dosage of 1 mg/kg body weight per day [45]. The HFD group and the coexposed group were fed a high-fat diet (60% Kcal as fat) until PND21.

### 2.2. Experimental Design

#### 2.2.1. Animals Housing

**Figure 1:**
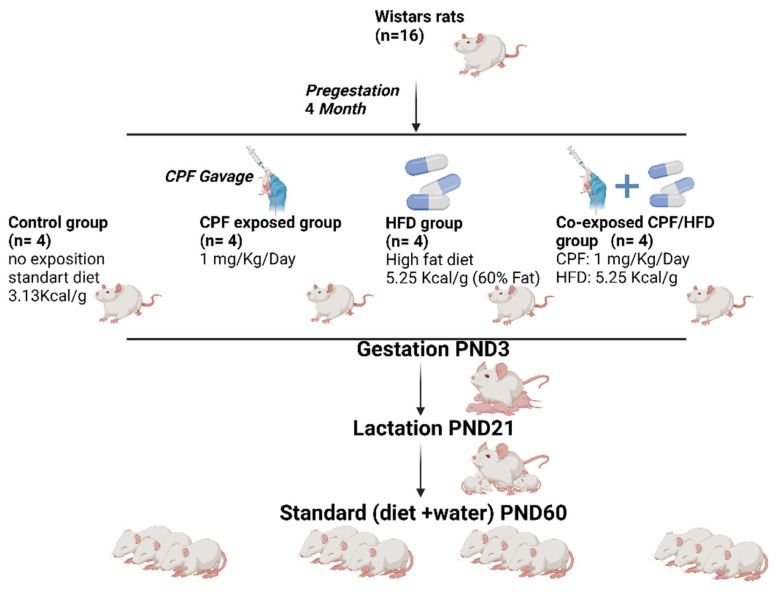
Study design and animal experiment.

A total of 16 female Wistar rats (Janvier Labs, Le Genest Saint Isle, France,), upon arrival, aged 7 weeks, were placed in cages and kept under constant conditions in a controlled environment. The room was maintained at a temperature of 23 °C to ensure a stable and regulated environment for the rats. Then, rats were housed in a NexGen Max cage system with an 81 in 2/523 cm^2^ floor area mounted on an EcoFlow rack system (Allentown Inc., Bussy Saint Georges, France). Following a week-long acclimation period, the female rats, with an average body weight of 225 ± 4.9 g, were randomly assigned to four groups. To facilitate social interactions, two rats were housed together in each cage. This housing arrangement allowed for appropriate socialization among the animals during the study (Figure 1).

#### 2.2.2. Ethical Approval and Euthanasia Method

The study protocol obtained approval from the Regional Directorate for Health, Animal and Environment Protection, an accredited organization in France. Additionally, it was also approved by the French Ministry of Research under the reference number APAFIS#8207-2016121322563594 v2. These regulatory approvals ensured that the study adhered to the necessary ethical and scientific standards regarding animal welfare and research conduct.

**Figure 2:**
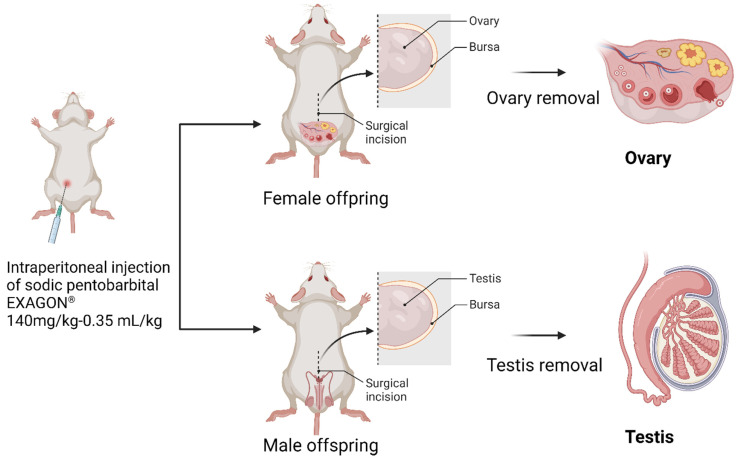
Offspring’s sacrifice and reproductive organ dissection.

At PND60, the offspring rats were euthanized using an injection of (1 mL·kg^−1^; 200 mg·mL^−1^ solution) sodium pentobarbital from EXAGON (Axience, Pantin, France). After euthanasia, the reproductive organs were dissected and placed directly into Krebs Henseleit solution from Sigma Aldrich (Saint Quentin Fallavier, France). This solution is commonly used to maintain the viability and functionality of isolated tissues during experimental procedures (Figure 2).

### 2.3. Immunohistochemistry (Kisspeptin and GnRHR)

Tissue sections were deparaffinized in xylene (Sigma Aldrich, Darmstadt, Germany) and rehydrated through decreasing concentrations of ethanol (TechniSolv, Paris, France). After deparaffinization, the slides were incubated in Tris EDTA buffer (10 mM tris-base (Cat no. 161-0719, Biorad, Hercules, CA, USA), 1 mM EDTA solution (Cat # 161-0729, Biorad, Hercules, CA, USA), and 0.05% tween20^®^ (Sigma-Aldrich, St. Louis, MO, USA), pH = 9) for 20 min (2 × 10 min) in a microwave (850 W) followed by washing with cold water for 10 min. To quench the endogenous peroxidase activity, the slides were incubated in water peroxide H202 (Gilbert) for 30 min then washed twice (each 5 min delicately) in TBS (ThermoScientific, Geel, Belgium) 0.025 Triton and blocked with 10% normal Goat Serum (ab7481) with 1% BSA (ThermoScientific, Waltham, MA, USA) in TBS for 2 h (C) between 0.5 and 10 μg/mL. Thereafter, the sections were incubated overnight in a humidity room at 4 °C with a primary antibody:

- For Kisspeptin detection: primary rabbit polyclonal anti Kisspeptin antibody (1:300 dilution, product no. 251265; Abbiotec. Escondido, CA, USA)
- For GnRHR detection: primary rabbit polyclonal to GnRHR (1:200 dilution; abcam, ab202848, Cambridge, UK)

The sections were washed in TBS/0.025 Triton 2 × (5 min) under gentle agitation and then incubated with goat anti-rabbit IgG-HRP (1:1000 dilution, product no. 252237; Abbiotec. Escondido, CA, USA) for 1 h at room temperature. After the washing step of 3 × (5 min) in TBS, labeling was visualized by incubating with a DAB (3,3′Diaminobenzidine) substrate kit (ab64238, abcam, Cambridge, UK) for 4 min and then washing in 5 min in TBS. Sections were counterstained with Mayer’s hematoxylin (VWR Q-Path Chemicals, Paris, France) for 2 min and mounted with Aqueous Mounting Medium (ab64230, Abcam, Cambridge, UK).

The slides were observed under a light microscope, specifically the Nikon ECLIPSE Ci model from Nikon Europe B.V. This microscope offers magnifications of ×4, ×20, and ×40, allowing for different levels of detailed observation. To capture the images, the NIS Elements version 1.10.00 imaging software was used. This software provides a platform for acquiring and processing images obtained from the microscope, enabling researchers to analyze and document their observations.

### 2.4. Histological Analysis

Histopathological analysis of the testicular and ovary sections (4 um thick) was performed using hematoxyline-eosine staining. There were 5 slides for each group, and each slide contained 2~3 sections of an organ.

The paraformaldehyde-fixed paraffin-embedded sections were incubated in Xylene 2 × (5 min) and then rehydrated using different concentrations of ethanol (2 min ethanol 100%, 2 min ethanol 95%, 6 min ethanol 70%, and 2 min osmosed water). The slides were stained with hematoxylin of Mayer 5% (2 min) and, after a washing step, for 2 min in osmosed water. The slides were soaked 5 times in acid/alcohol 37%. After the washing step, eosin 1% staining was realized for 1~3 min. Finally, the slides were washed and dehydrated (2 min ethanol 95%, 2 min ethanol 100%, and 2 min in xylene). Morphological analysis was performed under light microscopy (Nikon ECLIPSE Ci, Nikon Europe B.V) with ×4, ×20, and ×40 magnifications. The image was captured using an imaging software NIS Elements version 1.10.00.

The morphometric measurements were used to assess the number of cells per seminiferous tubule (average of 10 selected seminiferous tubules per section) and area of seminiferous tubules (average of 10 randomly selected seminiferous tubules per section) [46].

### 2.5. In Silico Quantification of Expression

The C, CPF, HFD, and CPF/HFD immunodetected sections were scanned and photographed using a Tissue Faxs plus system (Tissue Gnostics Medical & Biotech Solutions, Vienna, Austria). At least 5 randomly selected slides per animal were used for immunostaining and scanned. Regions of interest (ROIs) were manually delineated using the zooming and mark-up tools available in the TissueFAXS viewer. This process allows us to focus on specific regions for further analysis or measurements. The ROIs were then digitized at 20× using HistoQuest^®^ (TissueGnostics GmbH, Vienna, Austria), and the expression of Kisspeptin and GnRHR (the intensity of signals detected onto the sections) was quantified. A typical image was selected for presentation [47,48].

### 2.6. Statistical Analysis

Statistical data were analyzed using StatView software (version 5.0, SAS Institute Inc., San Diego, CA, USA). The results are presented as mean values ± standard deviations (SD), number (n), and percentage (%). The data exhibited a normal distribution, as confirmed by the Kolmogorov–Smirnov test of normality. We conducted a two-way ANOVA to investigate the main effects of diet (HFD and HFD+ CPF groups versus control and CPF groups) or CPF (CPF and HFD+CPF groups versus control and HFD groups) and to assess differences between the groups. In instances where an interaction between HFD and CPF was observed, we performed post hoc analyses using unpaired *t*-tests to compare individual groups. For all analyses, the significance threshold was set at *p* ≤ 0.05. The graphs were generated by GraphPad 8.0.1 software.

## 3. Results

### 3.1. Impacts of Treatment on Pups’ Body Weight

Statistical data analysis shows a significant increase in rat offspring’s body weight in the postnatal period *p* = 0.03. The high-fat diet group presents the heaviest body weight groups in the different PND periods.

The intergroup comparison between the control and the CPF-exposed group shows a highly significant decrease in the body weight in the CPF-exposed group at the period of PND30 and PND 60 (*p* < 0.05 and *p* < 0.001, respectively).

**Figure 3:**
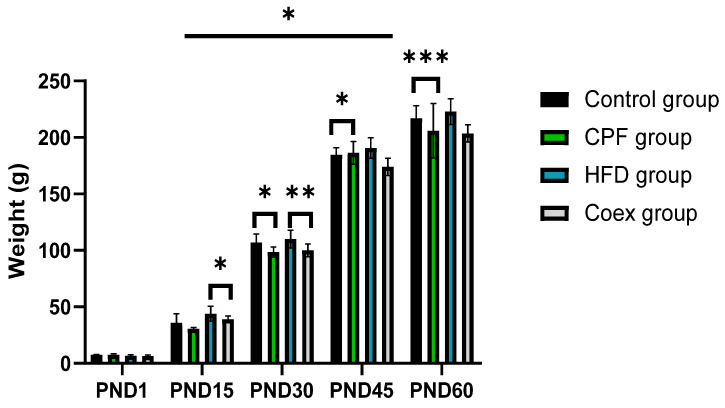
Evolution of the pups’ body weight (male + female) at the postnatal periods. Values are expressed as mean ± standard deviation (SD). *, **, and *** indicate significant difference (*p* < 0.05, *p* < 0.01, and *p* < 0.001, respectively). The statistical result was obtained using the analysis of variance (two-way ANOVA). CPF: Chlorpyrifos, HFD: High-fat diet, PND: Postnatal day, Coex: Coexposure.

At PND 15 and PND 30, the body weight of rat offspring shows a decrease in the group coexposed to CPF/HFD compared to the HFD group (*p* < 0.05 and *p* < 0.01, respectively); see Figure 3.

No significant differences were observed between the different groups at PND1, between the control group and CPF group at PND15, and between the HFD group and coex group at PND 45 and PND 60.

**Figure 4:**
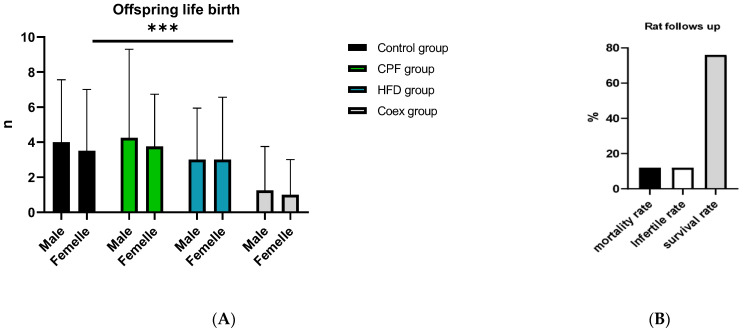
Rat follows up during the treatment (**B**) and gestational period (**A**). Values are expressed as mean ± standard deviation (SD). ***, significant difference at *p* < 0.001. The statistical result was obtained using the analysis of variance (two-way ANOVA). CPF: Chlorpyrifos, HFD: High-fat diet, Coex: Coexposure.

The outcomes of the rat study are shown in Figure 4, illustrating the count of male and female offspring within each group, alongside the mortality rate of the rats in Figure 4.

### 3.2. Histological Analysis

#### 3.2.1. Ovary of Rat Offspring

**Figure 5:**
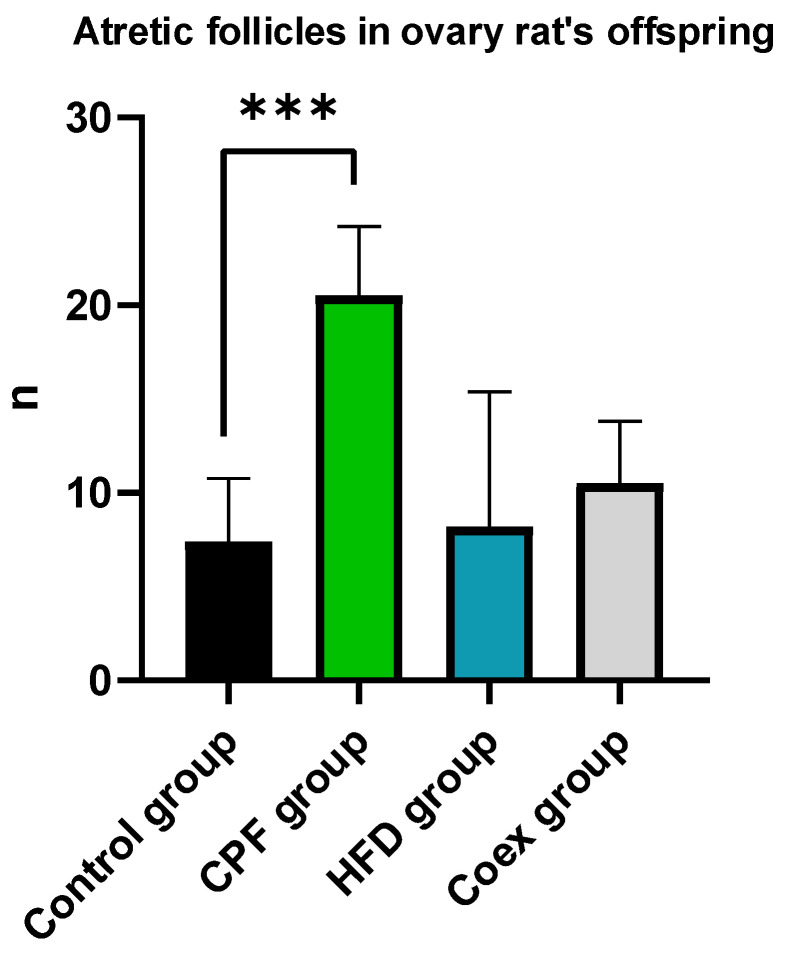
Measurement of atretic follicle number in the ovary of the rat offspring. Values are expressed as mean ± standard deviation (SD). *** show significant difference (*p* < 0.05, *p* < 0.01, and *p* < 0.001, respectively). The statistical result was obtained using the analysis of variance (two-way ANOVA). CPF: Chlorpyrifos, HFD: High-fat diet, Coex: Coexposure.

**Figure 6:**
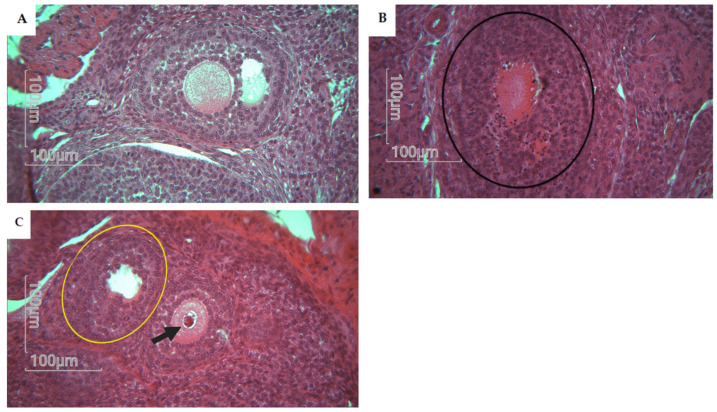
Histological analysis of rat offspring follicles showing alterations. Magnification ×40. (**A**) Represents a normal morphology of antral follicle with normal oocytes, normal cumulus cells, and theca cells. (**B**) Represents an atresia in follicle with color change to dark red absence of oocyte (black circle). (**C**) Represents a degenerative follicle, with no oocytes (yellow circle) and a follicle with fractured oocyte colored in dark red (black arrow).

Atresia in secondary and antral follicles was observed by eosin staining. Histological analysis of the rat ovary was performed in the control group (*n* = 5), CPF group (*n* = 5), HFD group (*n* = 5), and Coex group (*n* = 5). Statistical data show a significant difference in atretic follicle number between the CPF group and control group (*p* = 0.0008), as shown in Figure 5. Follicles with fractured oocytes were observed in some sections of the CPF-exposed group and Coex group, as shown in Figure 6.

#### 3.2.2. Testis of Rat Offspring

**Figure 7:**
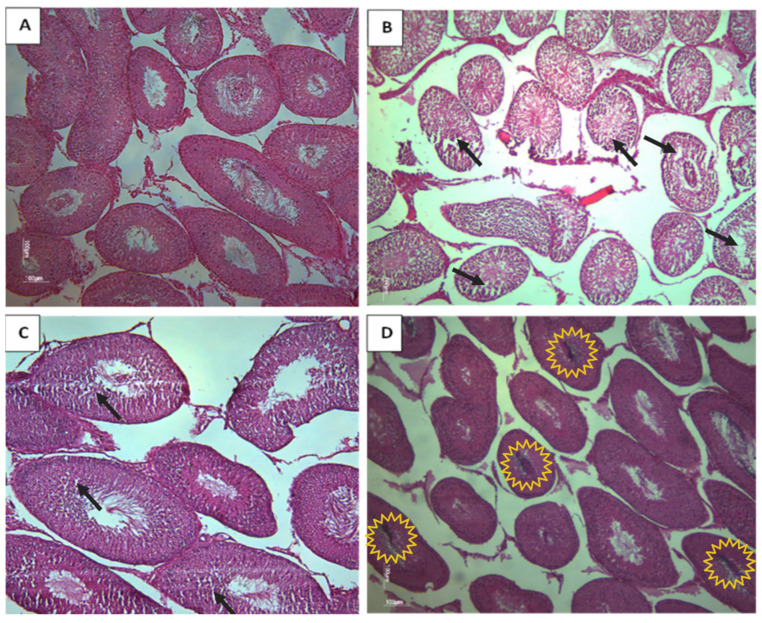
Histological testicular section of rat offspring exposed to different treatments (Magnification ×10). (**A**) Represents the normal morphology of rat testis. (**B**) Represents a testis morphology of CPF-exposed group. The observation reveals a disordered structure of STs, a notable absence of germ cells, and a partial reduction in the STs (black arrows). (**C**) Represents the HFD-exposed group; note the reduction in some germ cells (black arrows). (**D**) Represents the coexposed group, with the germ cells found in the tubular lumen (yellow explosion).

**Figure 8:**
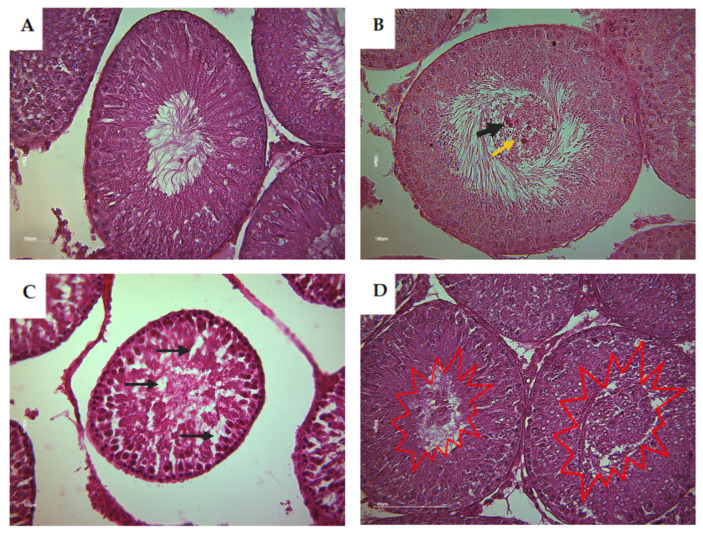
Histological detection of tubular section alteration of rat offspring (Magnification ×40). (**A**) Shows a normal ST containing various cell types including spermatogonia Ad and spermatogonia. (**B**) Shows primary spermatocytes and round and elongated spermatids with Sertoli cells and Leydig cells. (**B**) Shows the presence of some germinal lineage cells in the tubular lumen including primary spermatocytes (yellow arrow) and cells in degeneration (black arrow). (**C**) Shows an abnormal structure of ST with partial depletion or an absence of some germ cell (black arrow). (**D**) Shows an accumulation of a large quantity of germinal lineage cells in the tubular lumen including cells in degeneration, round spermatids, and primary spermatocytes (red explosion).

Based on the histological analysis of testis from rat offspring, the control group displayed typical and normal histological features of the seminiferous tubes (STs). However, in contrast, the CPF and the HFD subgroups exhibited a clear desquamation of the seminiferous epithelium (Figure 6). The Coex group presented a disorganized structure of STs (Figure 7D). The desquamation resulted in the release of cellular debris and a large number of germinal lineage cells, predominantly primary spermatocytes, cells in degeneration, and round spermatids presented in the tubular lumen (Figure 8B–D). 

Morphometric analysis of tubule sections did not exhibit significant statistical differences in the ST area between the different groups, as indicated in Table 1.

### 3.3. Kisspeptin and GnRHR Immunodetection

#### 3.3.1. Localization of Immunoreactive Kisspeptin and GnRHR in the Ovary of Rat Offspring

**Figure 9:**
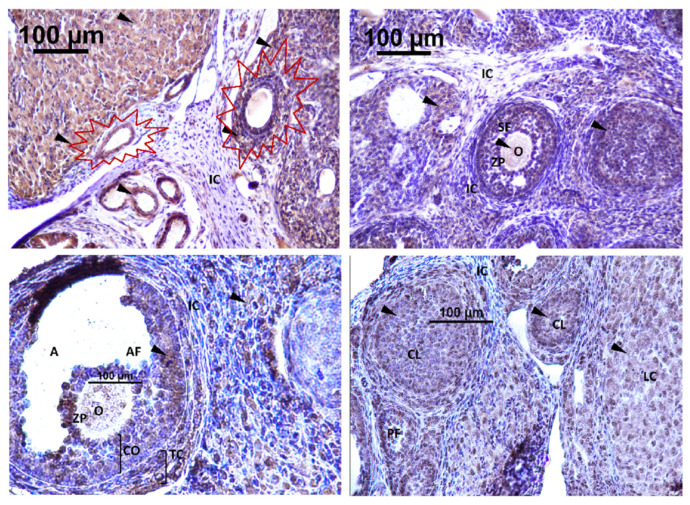
Immunodetection of Kisspeptin in rat ovary offspring. IC: interstitial cell, CL: corpus luteum cell, A: antrum, O: oocyte, ZP: zona pellucida, SF: secondary follicle, PF: primary follicle, AF: antral follicle, CO: cumulus oophorus, TC: theca cell, LC: luteal cell. The first section of the figure shows the atretic follicle (surrounded in red) at Magnification ×40. Immunodetection of Kisspeptin (triangle).

The expression of Kisspeptin was detected in different localizations within the rat ovary. Strong immunoreactivity for GnRH was exhibited in the CL, corpus luteum; LCs, luteal cells; O, oocyte; TC, theca cell of the atretic follicle; and the cytoplasm of GCs, granulosa cells. No detection was observed in interstitial tissue and the theca cells of the secondary follicle (Figure 9).

**Figure 10:**
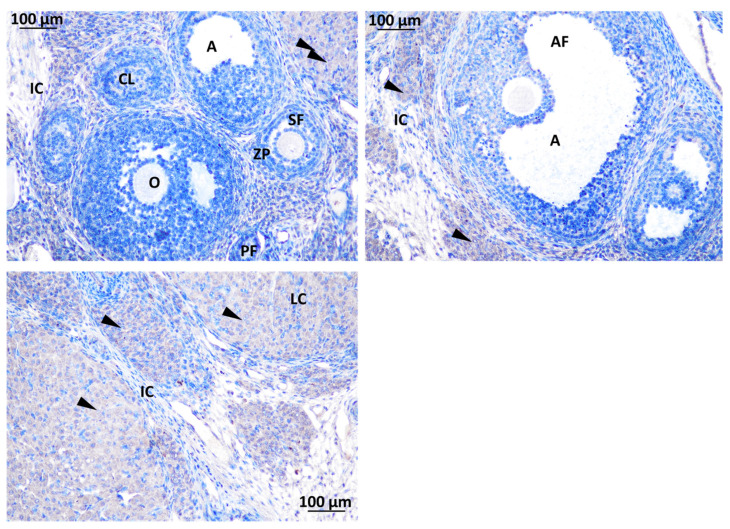
Immunodetection of GnRHR in rat ovary offspring; IC: interstitial cell, CL: corpus luteum, A: antrum, O: oocyte, ZP: zona pellucida, SF: secondary follicle, PF: primary follicle, AF: antral follicle, LC: luteal cell. Magnification ×40.

Strong immunodetection for GnRHR was noted in the interstitial cell (IC) and O, oocyte, within the rat ovary offspring (Figure 9). There was no detection of GnRHR in the SF, secondary follicle; PF, primary follicle; AF, antral follicle; CO, cumulus oophorus; TC, theca cell; and GC, granulosa cell (Figure 10).

#### 3.3.2. Localization of Immunoreactive Kisspeptin and GnRHR in the Testis of Rat Offspring

**Figure 11:**
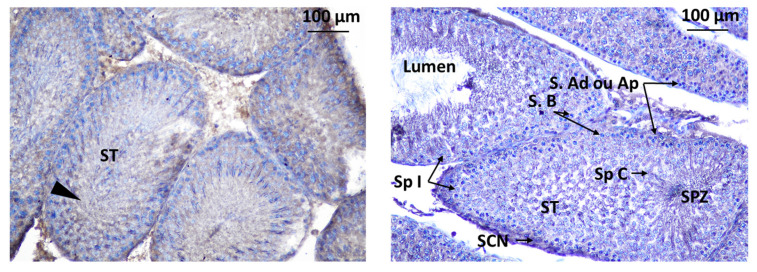
Immunodetection of Kisspeptin in seminiferous tubules of rat offspring’s testis; S.Ad: spermatogonia Ad, SpI: spermatocyte I, SCN: Sertoli cell nuclei, ST: seminiferous tube, S.B: Spermatogonia B, SpC: Spermatogonia C, SPZ: Spermatozoa, S.Ap: Spermatogonia Ap. Innunodetection of Kisspeptin (triangle).

Immunodetection of Kisspeptin revealed its presence in specific areas within the testis of rat offspring. Kisspeptin was observed in two main locations in the testis: interstitial Leydig cells (LCs) in the interstitial spaces between the seminiferous tubules, suggesting a potential role in regulating testosterone production, and inside the seminiferous tubules within the seminiferous tubules, indicating a possible direct influence on spermatogenesis (Figure 11).

**Figure 12:**
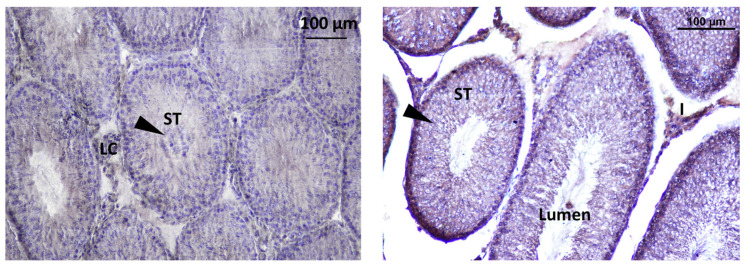
Immunodetection of Kisspeptin in seminiferous tubules of rat offspring’s testis; LC: Leydig cell, ST: seminiferous tube, I: intertubular.

The localization of GnRHR was observed in the interstitial Leydig cells (LCs) and inside the seminiferous tubule. The localization of immunoreactive Kisspeptin and GnRHR was observed in the testis of rat offspring (Figure 12).

### 3.4. In Silico Quantification of the Expression of Kisspeptin and GnRHR

The levels of Kisspeptin and GnRHR expression in the reproductive organs (testis and ovary) of rat offspring were measured using HistoQuest^®^.

**Figure 13:**
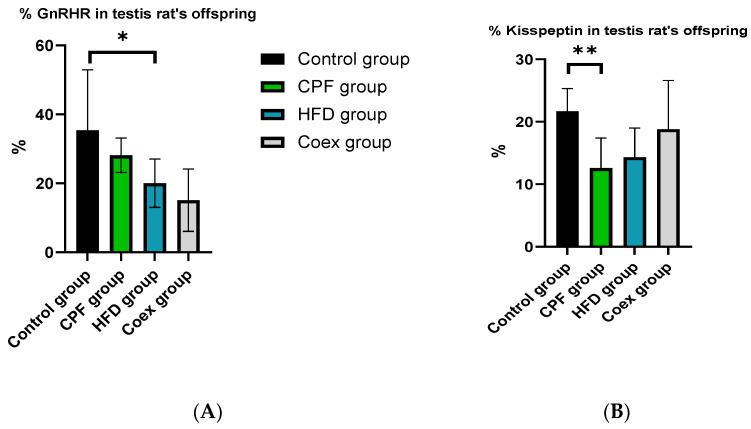
Effect of different treatments on GnRHR and Kisspeptin expression in testis of rat offspring. Values are expressed as mean ± standard deviation (SD). * and ** indicate significant difference (*p* < 0.05, *p* < 0.01, respectively). The statistical result was obtained using the analysis of variance (two-way ANOVA). CPF: Chlorpyrifos, HFD: High-fat diet, PND: Postnatal day, Coex: Coexposure. (**A**) represents the percentage of GnRHR in the testis of rats’ offspring and (**B**) represents the percentage of Kisspeptin in the testis of rats’ offspring.

The statistical analysis revealed a significant decrease in the percentage of GnRHR (*p* = 0.01) in the offspring from the high-fat diet (HFD) group compared to the control group. However, there were no significant differences observed between the control group, the group exposed to CPF (chlorpyrifos, a pesticide), and the coexposure group (*p* ≤ 0.05) (Figure 13A).

The data analysis demonstrated a significant reduction in the percentage of Kisspeptin (*p* = 0.002) in the testis rat offspring exposed to CPF compared to the control group. No significant difference was found in the other groups (*p* ≤ 0.05) (Figure 13B).

The quantification of levels of Kisspeptin and GnRHR in the ovary of rat offspring showed no significant difference between the different groups (*p* ≤ 0.05).

**Figure 14:**
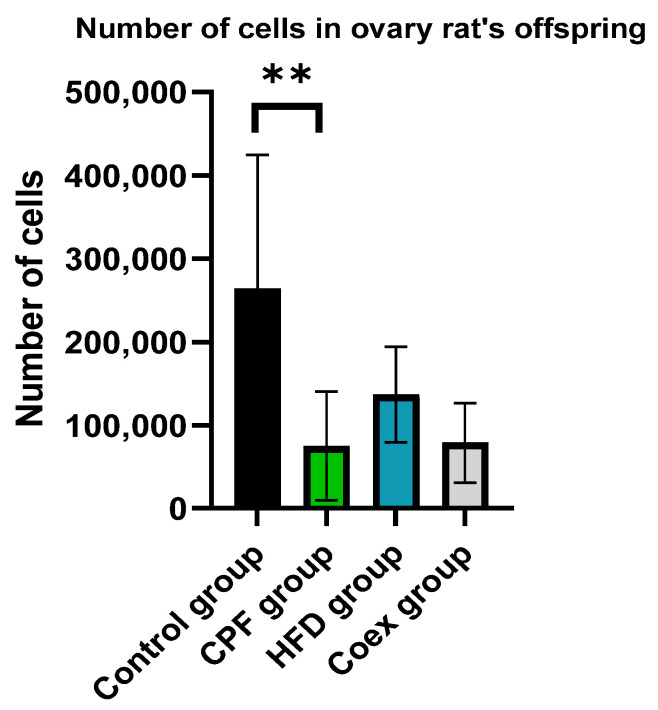
Effect of Different Treatments on the number of cells in ovary of rat offspring. Values are expressed as mean ± standard deviation (SD). **, significant difference at *p* < 0.001. The statistical result was obtained using the analysis of variance (two-way ANOVA). CPF: Chlorpyrifos, HFD: High-fat diet, Coex: Coexposure.

The comparison of the number of cells in the ovary of rat offspring revealed a significant decrease in cell number shown in the CPF group compared to the control group (*p* = 0.002). No significant difference was found in the other groups (*p* ≤ 0.05; Figure 14).

Data analysis of testis’ cell number in different treated groups showed no significant differences in cell number between the groups (*p* ≤ 0.05).

## 4. Discussion

The present study investigates the adverse effects of chlorpyrifos pesticide and high-fat diet on fertility and reproduction function in rat offspring. Chlorpyrifos pesticide was chosen in this study because it was widely used in agriculture, especially in France, and it is considered an endocrine disruptor [9,10]. In vivo studies have explored the effects of CPF treatment on body weight and birth weight; some have documented an increase in body weight and birth weight with CPF exposure, while others have shown a decrease [18,49].

The current study revealed a significant gain in body weight from PND1 to PND60 with the highest body weight observed in the HFD group. The intergroup comparison at PND30 and PND60 showed a significantly lower body weight in the CPF-treated groups compared to the other groups. Similar results were observed by Condette et al., 2014, who revealed that rats exposed to CPF exhibited a notable increase in weight (averaging 13 g; *p* < 0.01) and length (averaging 2 cm; *p* < 0.01) compared to non-exposed groups [50]. In addition, the body weight of rat offspring decreased in the coexposed (Coex) group compared to the HFD group at PND 15 and PND 30. This finding suggests the toxic effect of CPF. Several studies corroborate the observed results [18,45]. Similarly, Akhtar et al., 2009 revealed a significant body weight decrease at a high dose (9 mg kg^−1^d^−1^) of chlorpyrifos [12]. Conversely, Silva in 2021 reported that males exposed to CPF showed increased body weights [51]. Perinatal exposure to CPF causes indirect toxicity for rat offspring by leading to a hormonal disorder through the endocrine-disrupting property [9]. CPF can block or alter growth hormones, including GnRH, which contributes to a developmental decline [52]. The morphological analysis of rat testis showed an abnormal testis structure, including the depletion of seminiferous epithelium and some germinal lineage cells and their absence, especially in the CPF-exposed group and HFD-exposed group. A similar outcome was found in the study by Spooner in 2015 [53,54]. It seems that exposure to CPF may have detrimental effects on spermatogenesis and the production of viable sperm [46].

No significant differences in the seminiferous tube area were observed, but histopathological signs were detected. This alteration is defined by the presence of some germinal lineage cells in the tubular lumen, including primary spermatocytes and cells in degeneration, and an accumulation of large quantities of germinal lineage cells in the tubular lumen. The study by Gabriel et al., 2014 confirms the current results; they reported that oral ingestion of Permethrin led to abnormalities such as disruption of the normal architecture, decrease in mature sperm cells, reduction in luminal diameter, and reduced interstitial spaces [55]. Histological analysis of the ovary of rat offspring showed a significant increase in the atretic follicle in the CPF-exposed group compared to the control group. It seems that CPF disrupts folliculogenesis and leads to follicle and oocyte degeneration [56]. Several studies demonstrate similar results [57,58].

Our study demonstrated the immunodetection of Kisspeptin and GnRHR for the first time in the reproductive organs of rat offspring. This finding suggests the presence and potential role of Kisspeptin and GnRHR in the reproductive development of rats as reported in other studies [31,34,35,42]. In the ovary of rat offspring, the immunodetection of Kisspeptin was found in the different stages of follicles (from primordial to antral follicle) and in the corpus luteum cells. Indeed, the results of the study strongly suggest that Kisspeptin plays a significant role in the regulation of folliculogenesis and suggests its involvement in regulating the development and maturation of ovarian follicles [59]. Similarly, the immunodetection of Kisspeptin in the testis of rat offspring, including seminiferous tubes and Leydig cells, reveals the function of Kisspeptin in spermatogenesis, initiation of puberty, regulation of the male reproductive system, and overall control of testosterone secretion [30,60].

**Figure 15:**
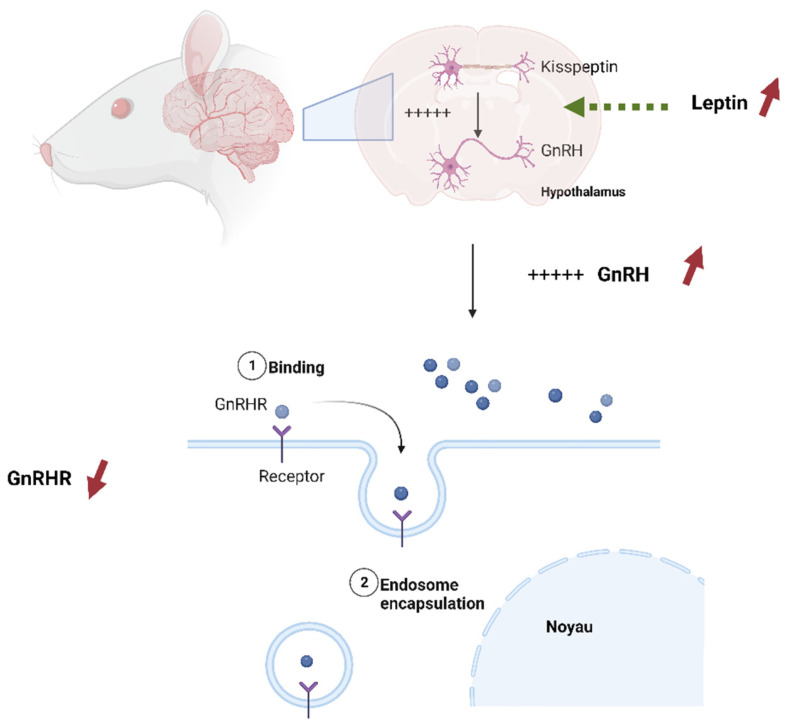
GnRHR downregulation as a response to High-fat diet, 1: the GnRH hormone binds in the the receptor (GNrhr), when the level of leptin increases, it leads to an increase in GnRH (red inscreased arrows) 2: The endosome encapsulates the GnRH-GnRHR complex in response to increasing GnRH (red decreased arrow).

This study is the first to assess the impact of pesticide exposure and HFD on Kisspeptin and GnRHR presence in the reproductive organs of rat offspring. Our findings demonstrate a reduction in the expression of GnRHR in the testis of rat offspring exposed to HFD. In the context of a high-fat diet, excessive intake of dietary fats can lead to an increased release of leptin, a hormone produced by adipose (fat) cells in response to the body’s energy reserves [61]. Leptin receptors are present on Kisspeptin neurons in the hypothalamus. When leptin binds to these receptors, it stimulates the activity of Kisspeptin neurons, leading to an increase in Kisspeptin expression. Kisspeptin, in turn, stimulates the release of GnRH from the hypothalamus [62]. However, over time, constant exposure to a high-fat diet leads to an increase in leptin expression and, as a result, an increase in GnRH [62]. It is widely known that the responsiveness of cells to specific signaling hormones includes different mechanisms to regulate homeostasis [63]. These mechanisms include receptor downregulation and receptor desensitization [64]. It seems that, as a response to excessive stimulation of GnRH after the mother’s high-fat diet exposure, testis cells applied a downregulation mechanism to reduce the number of GnRHR receptors (Figure 15).

Hiller-Sturmhöfel et Bartke, 1998, defined this process as the internalization of some receptors, effectively removing them from the cell surface [63]. As for CPF exposure, our results show a negative impact on the expression of Kisspeptin in the rat offspring’s testis. Chlorpyrifos, as an endocrine disruptor, alters the expression of extrahypothalamic Kisspeptin in rat’s testis. This result is similar to the study by Johanna who reported a decrease in Kisspeptin expression in cells exposed to dioxin [65,66]. Conversely, it was revealed that Kiss1 expression was not affected by endocrine disruptors [67].

In the ovary of rat offspring, the comparison of Kisspeptin and GnRHR does not exhibit persistent changes after perinatal exposure to CPF, HFD, and coex, whereas several studies have shown a negative link between pesticide exposure and Kisspeptin expression [30,36,68,69].

The strategy of this study is to use chronic low-dose perigestational exposure to understand the indirect effect on the progeny. Thus, our study highlights the concept of the “window of vulnerability” of the “Developmental Origin of Health and Disease” [17]. In other terms, exposure to food contaminants such as pesticides during the first 1000 days of life increases vulnerability to chronic diseases [70].

In the current study, the perinatal exposure of female rats to chlorpyrifos and/or a high-fat diet had no effect on the expression level of Kisspeptin and GnRH receptor in the female offspring; however, it significantly impacted the male offspring. The differential response of the male and female offspring to the exposure raises questions about the underlying mechanisms and biological differences between the sexes [71]. It may indicate that there are sex-specific pathways or hormone signaling networks involved in the regulation of Kisspeptin and GnRH receptor expression [72]. The DOHaD hypothesis suggests that early life exposures lead to the development of chronic diseases such as obesity, diabetes, cardiovascular diseases, and even certain neurodevelopmental disorders [22]. These periods of development (prenatal and early postnatal periods) are considered critical windows of vulnerability [20]. During these windows, cells and tissues are rapidly differentiating and developing, making them more susceptible to disruptions caused by environmental exposures [20]. Chronic low-dose exposure to CPF can have indirect effects on offspring when it occurs through maternal exposure during pregnancy [18]. CPF can cross the placental barrier, reach the developing fetus, and lead to an indirect health alteration of offspring, which can have long-term health impacts [73]. In addition, our findings suggest the implication of non-genetic mechanisms through “epigenetics” that might explain these changes in male offspring in response to the maternal environment during early development and the postnatal period [21]. Exposure to CPF during pregnancy can also influence gene expression patterns in the fetus through epigenetic mechanisms [21]. Understanding these sex-specific differences could be crucial for developing targeted interventions or therapies to mitigate the potential negative impacts of environmental factors on reproductive health [74].

A significant severe reduction in cell number was demonstrated in the offspring’s ovary of the rat exposed to CPF. It seems that the CPF induces oxidative stress in ovary tissue, leading to an increase in ROS levels within the ovarian tissue. These ROS are highly reactive and can cause damage to cellular components, such as lipids, proteins, and DNA. As a result, the delicate balance within the ovarian cells is disrupted. The apoptosis process is initiated as a protective response to eliminate cells that are severely affected by the ROS-induced damage [75,76,77,78].

## 5. Limitations and Perspectives

To investigate the impact of pesticide exposure and a high-fat diet on reproductive organ expression, we used an animal model. While it can provide valuable insights, there may be differences between rats and humans, making it necessary to interpret the results with caution when applying them to humans. The study exposes rats to a specific dose of the pesticide chlorpyrifos (CPF) and a high-fat diet (HFD). However, it is important to note that the chosen exposure levels may not accurately reflect real-world exposure scenarios for humans, which can vary significantly. In fact, exposure to pesticide residues is not limited to one pesticide but a mixture of organophosphates, carbamates, pyrethroids, etc. In consequence, it is of real importance to study the cocktail effect of the common pesticide residues we are orally exposed to and evaluate the impact compared to that of one pesticide [79].

We aim to use in silico analysis software to compare the expression of Kisspeptin and GnRHR in different groups. While this approach can provide valuable insights, it relies on computational models and may have limitations in accurately capturing the complexity of biological systems. Understanding the impact of pesticide exposure and dietary factors on reproductive organ expression may have implications for reproductive health. The findings of this study may contribute to the development of preventive strategies or interventions to mitigate the potential negative effects on reproductive function.

## 6. Conclusions

The present study reveals that perinatal exposure of female rats to chlorpyrifos and/or a high-fat diet induces a histopathological impact on reproductive organs (testis and ovary) in the offspring. Notably, the presence level of Kisspeptin and GnRH receptor in the testis of rat offspring declined in the CPF group and HFD group, respectively, indicating that the offspring’s testis is more sensitive to obesity and pesticide exposure than the offspring’s ovary. This study required us to better understand the link between environmental factors, obesity, and reproductive function.

## Figures and Tables

**Table 1:** Area (µm^2^) of seminiferous tubule sections observed in rat offspring in different groups; Control, CFP group, HFD group, and Coexp group.

	Mean ± Sd (µm^2^)	F Value	*p*-Value
Control group	1107.445 ± 781.704		
CPF group	1255.563 ± 636.659	0.2	0.6
HFD group	1360.484 ± 542.038	0.08	0.7
Coexp group	1082.892 ± 442.743	2.4	0.1

Values are expressed in mean ± standard deviation (Sd) and two-way ANOVA tests at a significance of (*p*-value) *p* ≤ 0.05. F-value: F-statistic.

## Data Availability

Not applicable.
